# Development of a Vascularized Skin Construct Using Adipose-Derived Stem Cells from Debrided Burned Skin

**DOI:** 10.1155/2012/841203

**Published:** 2012-07-05

**Authors:** Rodney K. Chan, David O. Zamora, Nicole L. Wrice, David G. Baer, Evan M. Renz, Robert J. Christy, Shanmugasundaram Natesan

**Affiliations:** ^1^Dental and Trauma Research Detachment, United States Army Institute of Surgical Research, Fort Sam Houston, TX 78234-6315, USA; ^2^Department of Extremity Trauma Research and Regenerative Medicine, United States Army Institute of Surgical Research, Fort Sam Houston, TX 78234-6315, USA; ^3^Burn Center, United States Army Institute of Surgical Research, Fort Sam Houston, TX 78234-6315, USA

## Abstract

Large body surface area burns pose significant therapeutic challenges. Clinically, the extent and depth of burn injury may mandate the use of allograft for temporary wound coverage while autografts are serially harvested from the same donor areas. The paucity of donor sites in patients with burns involving large surface areas highlights the need for better skin substitutes that can achieve early and complete coverage and retain normal skin durability with minimal donor requirements. We have isolated autologous stem cells from the adipose layer of surgically debrided burned skin (dsASCs), using a point-of-care stem cell isolation device. These cells, in a collagen—polyethylene glycol fibrin-based bilayer hydrogel, differentiate into an epithelial layer, a vascularized dermal layer, and a hypodermal layer. *All-trans-retinoic* acid and fenofibrate were used to differentiate dsASCs into epithelial-like cells. Immunocytochemical analysis showed a matrix- and time-dependent change in the expression of stromal, vascular, and epithelial cell markers. These results indicate that stem cells isolated from debrided skin can be used as a single autologous cell source to develop a vascularized skin construct without culture expansion or addition of exogenous growth factors. This technique may provide an alternative approach for cutaneous coverage after extensive burn injuries.

## 1. Introduction 

Burns are a significant problem in combat casualty care and severe thermal injury accounts for approximately 5% of all combat casualties [1, 2]. In 2011, the American Burn Association approximated that 450,000 people suffer from burn injuries requiring medical treatment, an increase of 340% since 1995 [3, 4]. Of these, 45,000 require hospital admission, and only 55% (24,750 admissions) will enter one of the 125 hospitals with specialized burn care centers [3, 4]. From a clinical standpoint, the total body surface area (TBSA) affected, depth of burn, location on the body, and nature of the burn injury determines the need for tissue-grafting, tissue substitutes, all of which directly affect the resulting morbidity and mortality.  In particular, as the TBSA increases, mortality increases proportionately as a result of the inability to achieve skin closure [5, 6]. Currently, large-body-surface-area burns pose significant therapeutic challenges with implications for early hemodynamic instability and sepsis, as well as later complications of scarring, contracture, and long-term disability. Patients generally receive definitive care from burn surgeons which includes escharotomy, escharectomy, debridement, grafting, and reconstruction [7–9]. From a clinical standpoint, the extent and severity of burn injury determines the need for tissue-grafting or tissue substitutes. Though autografting remains the treatment of choice for excised burn wounds, this option may be severely limited in patients with extensive burns because of limited donor site availability [10–12]. Furthermore, repeat harvesting of split thickness skin grafts from the same donor area can result in morbidity, loss of dermal thickness, excessive scarring, and increased pain. 

When the extent of a burn exceeds the ability to perform a single-stage autograft, many burn centers have adopted the use of an allograft as a temporizing measure while they await further availability of autografts [10–12]. Although a wide variety of skin substitutes have been developed [13–15], the time required for revascularization and for cell expansion has limited the clinical utility of this option. Presently, available skin substitutes can be divided into those that replace the epidermis or the dermis [15, 16]. Epidermal substitutes such as cultured epithelial autograft are only several cell layers thick and lack much of the normal dermal organization and are therefore prone to frequent breakdown and infection, even years later. While dermal replacements are available, (e.g., Integra, Alloderm) their usage is limited by the time required for incorporation and revascularization, therefore increasing their propensity for infection, desiccation, and eventual graft loss [17]. The addition of cellular elements to dermal scaffolds have demonstrated superiority with respect to revascularization, but the lack of an available and convenient autologous cell source from a severely burned patient has not made this practical. 

The requirements for an ideal substitute include minimal donor site morbidity, availability, and the ability to reconstitute the different functions and layers of skin. Products based on autologous cultured keratinocyctes and fibroblasts are more likely to contribute to actual skin restitution [18–20]. Still, challenges involved with these products are the need for culture expansion and the extensive cultivation time. Moreover, such products require donor biopsy of normal skin to obtain the requisite cell types when normal tissue accessibility has proportionately decreased with percentage increase in TBSA [16].

Alternatively, developing a tissue engineered skin substitute using stem cells proves to be a potential option to regenerate skin for the treatment of extensively burned patients [21]. In particular, adipose-derived stem cells (ASCs) have been shown to possess immense potential to regenerate skin because of their substantial plasticity to differentiate into multiple cell lineages [22]. Unfortunately, after severe burn injury, the source(s) of adipose tissue can be limited because of the availability of uninjured viable tissue and the fear of causing additional morbidity from subcutaneous liposuction. Because tangential debridement of skin often leads to debridement of some viable tissue, we have shown that stem cells can be isolated in adequate quantities from the adipose layer of discarded burn skin (dsASCs). Furthermore, these cells are able to integrate within the excision wound bed of an athymic rat [21]. Unlike other cell types, such as fibroblasts, keratinocyctes, and endothelial cells that definitively require culture expansion, dsASCs can be isolated in proportionally large numbers from patients with an increasing percentage of TBSA burn. We have previously shown that dsASCs possess multilineage differentiation ability. In this study, we demonstrate that dsASCs can be isolated from discarded burned skin obtained after debridement using a “good manufacturing practice” grade processing technique. These stem cells can then be used along with collagen and fibrin-based scaffolds to develop epithelial, dermal-vascular, and hypodermal layers, which can then be used to develop a complete full-thickness skin equivalent. 

## 2. Materials and Methods

### 2.1. Burn Patients, Surgical Procedure, and Allograft Usage

Under an approved protocol (# H-11-003), the burn registry at the US Army Institute of Surgical Research (USAISR) was queried to determine the number of active-duty service members who have sustained burn injuries, including the TBSA during Operation Iraqi Freedom and Operation Enduring Freedom. The hospital's electronic medical record system was used to obtain information about the number of operations performed and the use of cryopreserved allograft (CPA) skin.

### 2.2. Discarded Burn Tissue and Patient Population

Injured skin from burn patients that was discarded after burn wound debridement was obtained from the USAISR Burn Center. Tissue samples were collected in accordance with a protocol reviewed and approved by the US Army Medical Research and Materiel Command Institutional Review Board (no. HSC20080290N). Discarded skin samples were brought to the laboratory immediately after debridement and 5 mm punch biopsies were taken from the sample and either fixed in 10% neutral buffered formalin (NBF) for histological analysis or were cryopreserved by using gradient sucrose cryopreservation technique [23]. Briefly, the tissue biopsies were treated with 4% paraformaldehyde (PFA, EMS, Hatfield, PA, USA) for 20 minutes, washed with Hank's balanced salt solution (HBSS, Invitrogen, Carlsbad, CA, USA) and treated serially with increasing concentrations of sucrose (from 5% and 20%), and then incubated overnight with 20% sucrose (Sigma-Aldrich, St. Louis, USA). The sucrose-treated biopsies were embedded in a 20% Sucrose-Histoprep mixture (2 : 1) (Fisher, Pittsburgh, PA, USA). The embedded samples were frozen by immersing them in isopentane, cooled by liquid nitrogen and stored at −80°C for immunohistochemical analysis.

### 2.3. Stem Cell Isolation from Discarded Skin Tissue

The debrided skin samples were washed three to four times with HBSS to remove adherent blood clots. The hypodermal layer (10 to 12 g) was dissected, transferred to a petri dish, and finely minced with sterile scissors or manual lipoaspirate. Stem cells were isolated using the point-of-care Transpose RTSystem (InGeneron, Inc., Houston, TX, USA) according to the manufacturer's instructions. Briefly, the minced adipose tissue was transferred to a 50 mL conical tube containing 20 mL of lactated Ringer's (LR) solution. To this mixture, 2.5 mL of Matrase enzyme (InGeneron) was added and the tube inverted several times to mix the minced tissue and enzyme solution thoroughly. The tubes were then placed in the Transpose RTsystem processing unit (Figure 3(e)), and tissue was subjected to enzymatic dissociation. After 45 minutes, the dissociated tissue was filtered and washed, and cells were pelleted in the Transpose RTsystem. The cell pellet was washed twice with phosphate-buffered saline and the resulting stromal vascular fraction (SVF) recovered. The SVF cell pellet was resuspended in growth medium (MesenPRO RS basal medium), supplemented with MesenPRO RS growth supplement, antibiotic-antimycotic (100 U/mL of penicillin G, 100 *μ*g/mL of streptomycin sulfate, and 0.25 *μ*g/mL of Amphotericin B), and 2 mM of L-glutamine (Invitrogen). Cells (1.5 to 2.0 × 10^6^) were seeded in a T75 tissue culture flask (BD Falcon, NJ, USA) and maintained in an incubator humidified with 5% carbon dioxide (CO_2_) at 37°C. After 4 hours in culture, the growth medium was replaced in the flasks to remove any floating debris. The remaining attached cells are designated as dsASCs.

### 2.4. Histological Methods

Histological analysis was performed on the 10%-buffered formalin-fixed discarded skin biopsies. The fixed samples were paraffin-embedded, and 5- to 7-*μ*m sections were cut and stained with Masson's trichrome stain (MTS) and/or hematoxylin and eosin (H&E). A “blinded” trained pathologist analyzed the MTS and/or H&E stained sections of the discarded human skin biopsies to assess tissue viability and burn depth.

### 2.5. Immunohistochemical and Immunocytochemical Analysis

Frozen sections (~8 to 10 *μ*m) of discarded skin biopsies, collagen gels with dsASCs differentiated to keratinocyctes, and polyethylene glycol (PEG)-fibrin-collagen-dsASCs bilayer gels were cut with a freezing microtome (Leica Microsystems, Nussloch, GmbH) and lifted onto glass slides. The sections were washed once with HBSS and fixed with 4% PFA for 20 minutes at room temperature. Nonspecific Fc receptor-mediated sites were blocked by incubating the sections for 2 hours with 1% bovine serum albumin (BSA) or 5% donkey serum in HBSS. The sections of discarded tissue, collagen gel with dsASCs differentiated to keratinocyctes, and bilayer gels with dsASCs were then incubated at 4°C overnight with antihuman monoclonal primary antibodies—platelet-derived growth factor beta (PDGFR*β*, 10 *μ*g/mL, BD Bioscience, San Jose, CA, USA), pan cytokeratin (5 *μ*g/mL, Abcam, Cambridge, MA, USA), 10 *μ*g/mL of neuron glial antigen 2 (NG2) (Billerica, MA, USA), and STRO-1 (10 *μ*g/mL, R&D Systems, Minneapolis, USA), respectively. The unconjugated primary labeled sections were washed twice (5 minutes) with HBSS and incubated with 5 *μ*g/mL of Alexafluor 488 or Alexafluor 594 labeled secondary antibodies of the corresponding Ig isotype for 45 minutes at 4°C. The nuclei were stained with Hoechst 33342 at a concentration of 10 *μ*g/mL for 20 minutes at room temperature (Invitrogen, Life Technologies, Grand Island, NY, USA). Nonspecific fluorescence was determined by using tissue sections incubated with respective fluorophore-labeled secondary antibodies alone.

Immunocytochemistry was performed on P1 dsASCs, cultured on a two-well chambered slide (20,000 cells/well) (Nalgene Nunc, LabTek Chamber Slide, Noperville, IL, USA) for 48 hours maintained in a 5% CO_2_ humidified incubator at 37°C. The cells were washed twice with HBSS, fixed with 4% PFA for 20 minutes at room temperature. The cells were then incubated overnight at 4°C with 10 *μ*g/mL of mouse antihuman PDGFR*β* monoclonal primary antibody (BD Bioscience). The cells were then incubated with secondary antibodies with fluorescently labeled IgG1 Alexafluor 594 labeled secondary antibody and Hoechst 33342 as described above.

### 2.6. Flow Cytometry

Passage 1 dsASCs were washed twice with HBSS, trypsinized and resuspended in fluorescence activated cell sorting (FACS) cell staining buffer (Biolegend, San Diego, CA, USA) to a final concentration of 5 × 10^5^ cells/100 *μ*L. Cells were immunostained with labeled primary mouse antihuman PDGFR*β*-PE (10 *μ*g/mL, BD Biosciences, San Jose, CA, USA) for 45 minutes. After incubation, the cells were washed twice and then resuspended in 500 *μ*L of cell staining buffer. FACS analysis was performed using a FACS Aria flow cytometer (Becton, Dickinson and Company, Mountain View, CA, USA). Prior to analysis, the forward scatter channel (FSC) and side scatter channel (SSC) properties were determined for each sample using appropriate unstained cells to eliminate dead cells and cell debris. Autofluorescence signals were eliminated by adjusting the signal outputs from designated channels, and the sensitivity was adjusted to collect a gated population of cells. Total percentage of cells staining positive for individual markers from the gated population was determined. Results were quantitated by FACS Diva software (BD Biosciences, Mountain View, CA, USA). Percent positive stem cells within the gated population were analyzed by collecting 20000 events.

### 2.7. Epithelial Differentiation

Type 1 collagen from rat tail tendon (5 mg/mL) was obtained from Travigen (Gaithersburg, MD, USA) and fibrillated according to the manufacturer's instructions by adjusting the pH to 6.8–7.0 using 100 *μ*L of Dulbecco's phosphate buffered saline (DPBS, Sigma-Aldrich) and 23 *μ*L of 1N sodium hydroxide (NaOH) per mL of collagen solution. The fibrillated collagen was added to culture plate inserts with an 8 m pore size membrane (six-well format, BD Falcon) and incubated for 30 to 40 minutes at 37°C. Following complete gelation of the collagen matrix, passage 2 (P2) dsASCs (75,000 cells/gel) were seeded over individual collagen gels and incubated for 36 hours with MesenPRO RS Basal Medium supplemented with MesenPRO RS Growth Supplement, antibiotic-antimycotic, and L-glutamine (Invitrogen) in a 5% CO_2_ humidified incubator at 37°C. Following this culture period, gels were switched to low glucose Dulbecco's modified minimal essential media (L-DMEM, Invitrogen) containing 5% fetal bovine serum (FBS, Invitrogen) supplemented with all-trans-retinoic acid (ATRA, 1 *μ*M/mL, Sigma-Aldrich). Four days after ATRA treatment, fenofibrate (75 *μ*M/mL, Alexis, San Diego, CA, USA) was added; 24 hours later, the gels were exposed to air (air-lifting) by removing the media within the inner chamber of the cell culture insert. Passage 2 dsASCs cultured over collagen gels under similar conditions without any inducers were used as no treatment controls. All the collagen gels were observed for 12 days, and light microscopic pictures were taken on days 4 and 12 with an Olympus IX71 inverted microscope (Olympus America, Center Valley, PA, USA).

### 2.8. Dermal-Vascular Differentiation

To develop the vascularized dermal layer, a collagen-PEGylated-fibrin gel construct was developed according to our previously described procedure [24] with slight modifications. Briefly, P2 dsASCs (50,000 cells/mL of gel mixture) were trypsinized and mixed with type 1 collagen (5 mg/mL; Trevigen, Gaithersburg, MD, USA) and fibrillated as described above. The collagen-dsASCs mixture was added to a six-well culture insert and incubated for 30 min in a 5% CO_2_ humidified incubator at 37°C to complete gelation. To prepare the PEGylated-fibrin-dsASCs layer, PEG-fibrinogen solution mixture was incubated for 20 minutes at 37°C. After incubation, dsASCs (50,000 cells/mL of gel mixture) and thrombin were added to the PEG-fibrinogen, and the solution mixture was carefully transferred on to the top of the collagen-dsASCs gels. The entire gel preparation was incubated for 10 minutes in a 5% CO_2_ humidified incubator at 37°C to obtain a collagen-PEGylated-fibrin-dsASCs bilayer hydrogel construct. The bilayer gels were incubated with alpha minimal essential media supplemented with 10% FBS, antibiotic-antimycotic (100 U/mL of penicillin G, 100 *μ*g/mL streptomycin sulfate, and 0.25 *μ*g/mL amphotericin B), and 2 mM L-glutamine (Invitrogen) and maintained in a 5% CO_2_ humidified incubator at 37°C. The stem cells within the gels were observed for 12 days, and light microscopic pictures were taken at days 6 and 12 with an Olympus IX71 inverted microscope.

### 2.9. Adipogenic Differentiation

Collagen-dsASCs (50,000 cells/mL of gel mixture) gels were prepared as described above. The cells were induced with adipogenic differentiation medium composed of DMEM with 10% FBS, 2 mM L-glutamine, and antibiotic-antimycotic (Invitrogen), supplemented with 1 *μ*M of dexamethasone, 200 *μ*M of indomethacin, 10 *μ*M of insulin, 0.5 *μ*M of isobutylmethylxanthine (IBMX) (Sigma-Aldrich, St. Louis, MO, USA), and were maintained in a 5% CO_2_ humidified incubator at 37°C and observed for 14 days [25]. To observe the staining of neutral lipids in the differentiated cells, the collagen gels were rinsed with HBSS, fixed with 4% PFA, and stained with Oil Red O. Undifferentiated dsASCs in collagen matrix served as controls. Light microscopic pictures were taken at days 7 and 14 with an Olympus IX71 inverted microscope.

## 3. Results 

### 3.1. Burn Wound Severity and Debridement

**Figure 1:**
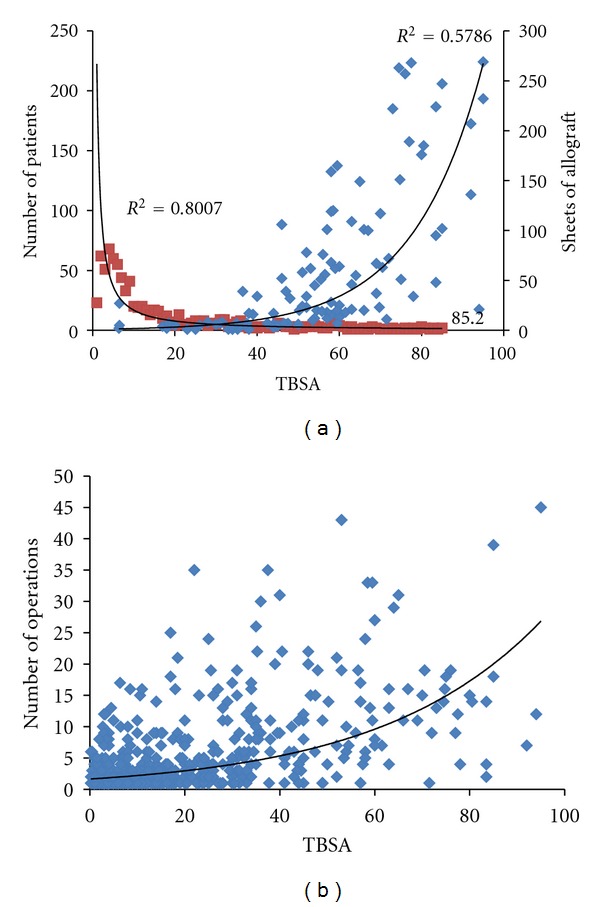
(a) Burn incidence superimposed onto the concurrent use of skin allograft in relation to TBSA. (b) Number of operations performed during the acute burn hospitalization as a function of TBSA. TBSA: total body surface area; CPA: cryo-preserved allograft.

Military service members treated at the USAISR burn center were identified through the burn registry. The number of operations performed, TBSA, and use of CPA for temporary wound coverage of 844 military patients over a period of 10 years were analyzed. The age distribution of burns within this population has been previously described [26] and Figure 1(a) represents their relationship to TBSA and probable incidences of CPA usage. The most burn admissions are concentrated towards lower TBSA range (<30%) while the majority of CPA usage is seen when TBSA is greater than 40%, which highlights the need for both temporary and permanent skin substitutes when donor sites are scarce. The surgical burden substantially increased as TBSA burns increased (Figure 1(b)), which inevitably requires escharotomy of necrotic and nonviable tissue and grafting using autologous skin, allografts, and skin substitutes, which are part of the standard clinical burn wound treatments.

**Figure 2:**
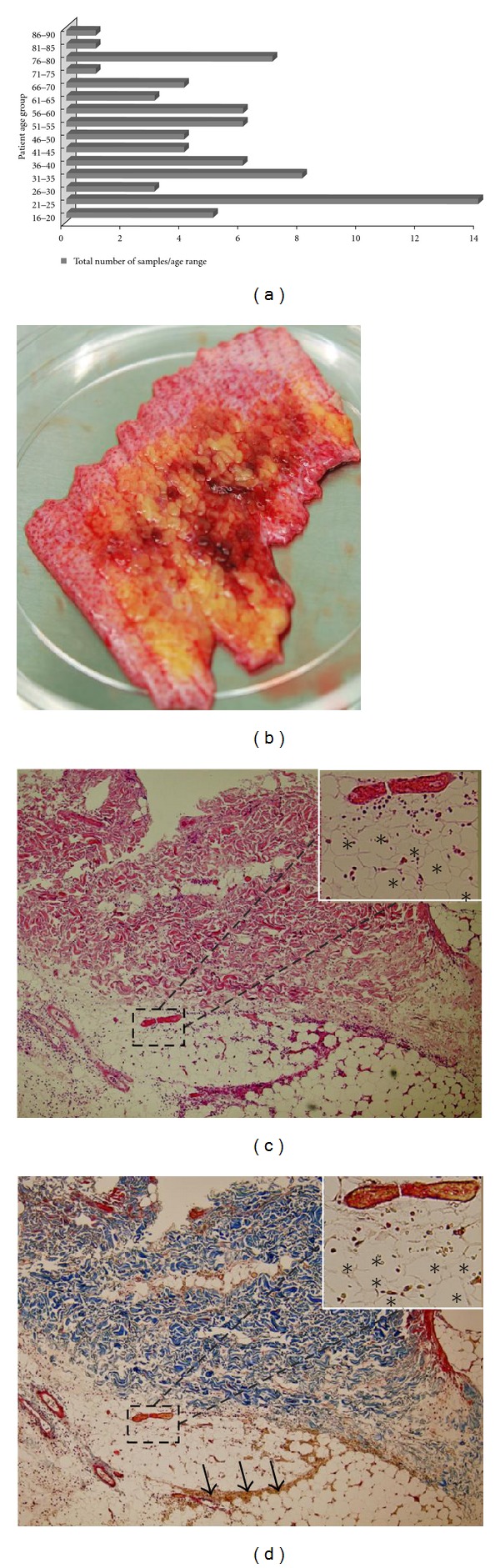
(a) Total number of discarded skin samples collected per age group for the patient population included in this study. (b) Discarded burn tissue. (c) Hematoxylin and eosin staining of burn tissue showing the loss of epidermal and dermal layers of the discarded burn skin. The inset figure is the magnified field of the section showing presence of collapsed blood vessel within the hypodermal layer (asterisks). (d) Masson's trichrome stained adipose tissue sections of discarded burn tissue showing collapsed and viable hypodermis (arrows). The figure inset is the magnified field view of the viable hypodermis (asterisks). Original magnification: ×100 (c, d) and ×400 (figure insets).

The discarded skin tissue after burn wound debridement also contained some viable wound beds, including the hypodermis, which was subjected to further analysis. In the present study, a total of 75 discarded skin samples were collected from patients varying in age from 16 to 90 (Figure 2(a)) with a mean age of 45. The discarded burn tissue (Figure 2(b)) had regions of both necrotized and viable adipose tissue. Histological analysis of the biopsied samples stained with H&E (Figure 2(c)) showed complete necrosis of epidermal and dermal region with infiltration of inflammatory cells and loss of vascular patency (inset). MTS sections of the debrided burn skin samples (Figure 2(d)) showed altered morphological appearance of the dermis with widespread hyalinization of collagen bundles. Both partially intact (asterisk, inset) and collapsed tissue layer (arrows) with granular cytoplasm and notable loss of clear vacuoles were observed within the adipose tissue. 

### 3.2. Stem Cell Isolation Process

**Figure 3:**
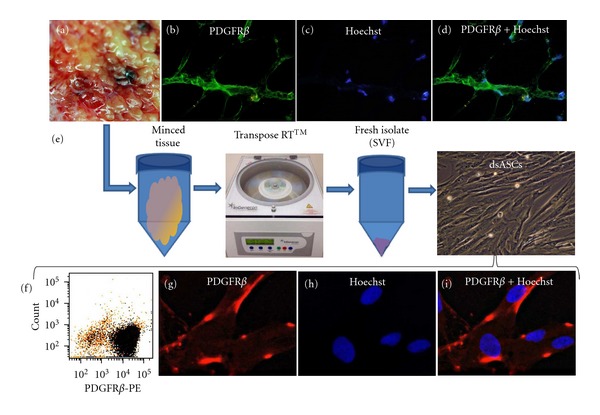
(a) Discarded burn skin. (b–d) Immunohistochemical images of section from discarded skin biopsies: (b) stained with PDGFR*β* (Alexa fluor 488), (c) Hoechst for nuclei, and (d) overlay of PDGFR*β* and Hoechst. (e) Stem cell isolation process from the adipose tissue layer of discarded burn skin (dsASCs) using the Transpose RTtissue processing system. (f) Fluorescent-activated cell sorting (FACS) analysis of P1 dsASCs stained with phycoerythrin-labeled PDGFR*β*. (g–i) Immunocytochemical images of P1 dsASCs: (g) stained with PDGFR*β* (Alexafluor 594), (h) Hoechst for nuclei, and (i) overlay of PDGFR*β* and Hoechst. Original magnification: ×200 (b–d and dsASCs in (e)) and ×600 (g–i).

The discarded tissue (Figure 3(a)), which was to be subjected to the cell isolation process, was first characterized for the presence of stem cells *in situ*. The debrided tissue stained positive for PDGFR*β* (Figure 3(b), stained green with Alexafluor 488), indicating the presence of resident stem cell population within the hypodermal adipose tissue layer. Figure 3(c) shows total cell nuclei stained (Hoechst) within this region, and Figure 3(d) shows an overlay of PDGFR*β* and nuclei. 

Cell isolation of the viable adipose tissue using the Transpose RTtissue processing system (Figure 3(e)) yielded a heterogeneous population of cells, SVF consisted of a mixture of mononuclear cells, and CD45^+^ blood-related cells. After isolation, the SVF was plated and the adherent cell population isolated and characterized. The SVF, when resuspended and plated in a tissue culture plate overnight, yielded *≈*7 to 9 × 10^5^ adherent cells/10 gm of adipose tissue processed (data not shown). FACS analysis of passage 1 (P1) dsASCs showed that more than 80% of the adherent cell population (5 × 10^5^ dsASCs) were PDGFR*β* ^+^. Furthermore, P1 dsASCs grown in chamber slides for 24 to 48 hours stained positive for PDGFR*β* (Figure 3(g), stained red with Alexafluor 594), demonstrating that the cells isolated from the adipose layer of the debrided tissue are of the same stem cell population originally identified *in situ* (Figure 3(c)). Figure 3(h) shows cell nuclei stained with Hoechst, and Figure 3(i) shows an overlay of cell nuclei and PDGFR*β*. 

### 3.3. Development of the Layers of Skin Substitute Using dsASCs

**Figure 4:**
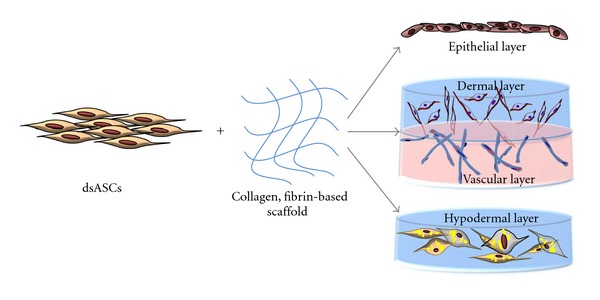
Development of different layers of skin substitute using dsASCs and hydrogel-based matrices. Epithelial and hypodermal constructs are developed using collagen hydrogel and the vascularized dermal construct using collagen-PEGylated-fibrin-based bilayered hydrogel.

Figure 4 depicts the strategy adopted to develop a skin substitute. In this process, dsASCs in combination with a collagen hydrogel were used to develop an epithelial and a hypodermal layer. Simultaneously, to reconstruct a vascularized dermal layer, a collagen-PEGylated-fibrin bilayer hydrogel construct was implemented. 

### 3.4. Epithelial Construct

**Figure 5:**
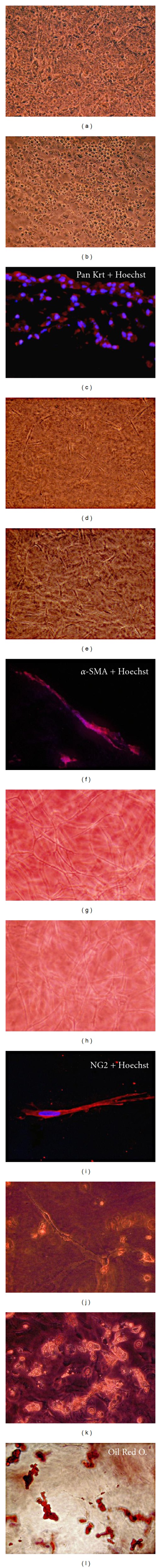
(a and b) Differentiation time course of dsASCs into epithelial-like cells on a collagen hydrogel showing (a) squamous cell-like morphology by day 4 and (b) epithelial-like cuboidal cell morphology by day 12. (c) Immunocytochemical image of section from dsASCs differentiated into epithelial-like cells on collagen gel (day 12) stained with pan cytokeratin. (d, e, g, and h) Differentiation time course of dsASCs in PEGylated-fibrin-collagen bilayer gels exhibiting bidirectional differentiation into fibroblast-like morphology in collagen layer (d; day 6) and tubular structures (g; day 6) in the PEGylated-fibrin layer. By day 12, collagen layers showed an increase in fibroblast-like cells (e) and complex networks in PEGylated-fibrin layers (h). Immunocytochemical image of section from bilayered hydrogel (day 12) depicting dsASCs to maintain stromal phenotype with collagen layer, stained with *α*-smooth muscle actin (f) and differentiated to vascular phenotype, stained with NG2 (i). (j and k) Differentiation time course of dsASCs into adipocytes on collagen showing appearance of oil vesicles by day 7 (j) and significant accumulation of oil droplets over time (k: day 14). (l) Oil Red O. staining of differentiated dsASCs (day 14) confirming formation of mature adipocytes within collagen gel. Bright field images original magnification: ×100 (a–h and l) and ×200 (j, k). Immunofluorescence original magnification ×400 (c, f, and i).

To develop the epithelial construct, dsASCs were seeded on top of a collagen hydrogel and initially induced with ATRA. The dsASCs seeded over the collagen matrix started to align into squamous cell-like morphology by day 4 (Figure 5(a)) and, after air-lifting and fenofibrate induction, were able to differentiate into epithelial-like cuboidal cell morphology by day 12 (Figure 5(b)). Immunocytochemical analysis of frozen sections of the epithelial differentiated dsASCs on the collagen gel showed positive staining for pan cytokeratin (red, Figure 5(c); nuclei are blue).  These results indicate that dsASCs could be differentiated into epithelial-like phenotype on a collagen matrix without using growth factors.

### 3.5. Vascularized Dermal Construct

A vascularized dermal construct was developed by seeding dsASCs within a collagen-PEGylated-fibrin bilayer hydrogel. This strategy allows for a simultaneous, bidirectional differentiation of dsASCs within the bilayer construct. The dsASCs exhibited fibroblast-like morphology within the collagen layer by day 6 (Figure 5(d)); and by day 12, the dsASCs remained fibroblast-like and had proliferated and populated the entire gel (Figure 5(e)). In contrast, within the PEGylated-fibrin layer of the bilayered gel, the dsASCs formed distinct tubular networks by day 6 (Figure 5(g)) and eventually formed dense networks by day 12 (Figure 5(h)). Immunostained sections of the day 12 bilayered gels showed presence of *α*-smooth muscle positive cells (Figure 5(f)) within the collagen layer indicating maintenance of stromal phenotype by dsASCs. Tubular networks within the PEGylated-fibrin layer stained positive for NG2, indicating PEGylated-fibrin gel supports dsASCs differentiation toward a pericyte lineage (Figure 5(i)). These results show that dsASCs within the bilayer hydrogel may be used as vascularized dermal equivalent.

### 3.6. Hypodermal Construct

The hypodermal construct was developed by differentiating dsASCs within a collagen gel using adipogenic differentiation media. Early during the induction of differentiation, the dsASCs proliferate within collagen gel; but only a few cells exhibited the presence of oil vesicles by day 7 (Figure 5(j)). However, by day 14, the dsASCs showed a significant accumulation of lipid droplets (Figure 5(k)). Lipid accumulation by the differentiated dsASCs within the collagen matrix on day 14 is shown with the positive staining with Oil Red O. (Figure 5(l)) confirming the commitment of dsASCs to form mature adipocytes within the collagen matrix.

## 4. Discussions

In this study, we have shown that stem cells isolated from discarded tissue can be used as a single viable cell source for epithelial, stromal, vascular, and adipose cells for the development of an autologous skin equivalent. These cell types in combination may allow for reconstitution of any or all of the individual layers of excised skin, including the epidermis, vascularized dermis, and hypodermis. The use of split-thickness skin graft is today's standard in the coverage of acute burn wounds after escharectomies. It provides reliable coverage of a large surface with relatively limited donor morbidity. However, when the TBSA is greater than 40%, donor sites become the limiting factor and preclude single-stage coverage with autograft alone. In this situation, an allograft is used for temporary coverage while donor sites are allowed to reepithelialize before repeat harvesting is possible. This technique, although standard, adds an approximate 2 weeks to the treatment timeline. 

The ability to use excised fat as a cell source, in combination with an extracellular matrix scaffold to regenerate the various layers of skin, has an inherent advantage of achieving coverage without the limitation of donor site availability. While our current method of using temporizing allograft can lead to successful coverage, the increased number of operations as well as time in the intensive care unit adds to the overall length of stay in the hospital. Furthermore, the use of split-thickness autograft does not actually replace all of the missing dermis or any of the hypodermis. This directly leads to fragile, nonpliable grafted skin for the patient. The ability to reconstitute all the layers of excised skin adheres to the sound principle that “form and function” are inextricably linked.

 Our group has previously shown that the discarded burn skin contains epidermis and dermis along with portions of the hypodermal adipose tissue deemed nonviable because of necrotized vasculature. However, this discarded tissue also contains viable perivascular structures. We have shown previously that this perivascular niche contains viable population of PDGFR*β* ^+^ cells [21]. Mounting evidences show that ASCs occupy the perivascular region [27, 28] and can be isolated and expanded as a rich population of multipotent stem cells [29–31]. ASCs have generally been isolated from lipoaspirates of healthy donors [32, 33]; however, in cases of extensive burn injury, liposuction may increase infectious complications of an already traumatized and immunologically compromised patient. Therefore, isolation of stem cells from adipose tissue layers of debrided burn skin is a practical way to obtain an adequate quantity of stem cells for reconstructive applications. The Transpose RTsystem used in this study to isolate dsASCs is a specialized device that provides a “good manufacturing practice” grade enzymatic tissue processing protocol that can be used in an operating room. More than 80% of the adherent dsASCs were PDGFR*β* ^+^, demonstrating the feasibility of obtaining stem cells by using the Transpose RTprocessing system. 

To reconstitute the three layers of skin using dsASCs, we utilized the inherent cues of three-dimensional matrix microenvironments consisting of collagen and PEGylated-fibrin-based hydrogel matrices. At this time, epidermal substitutes are usually developed by culture expanding normal human keratinocyctes from skin biopsies to develop cell sheets [16, 34–36] or cell sprays [37, 38]. The main drawback of these approaches, however, is the amount of time required (*≈*3 to 4 weeks) for culture expansion and the need to use special culture media and growth factors. In our present approach, chemical inducers approved by the US Food and Drug Administration (fenofibrate and ATRA) are used to differentiate dsASCs on a collagen hydrogel into epithelial-like cells within 12 days. The differentiated cells expressed pan cytokeratin and stratified keratin markers specific for epidermal keratinocyctes (unpublished data). Using this same approach, dsASCs may also be used in conjunction with other available collagen-based matrices to develop an epithelial substitute. 

One of major reasons for the failure of existing dermal or skin equivalents is the inability to become revascularized within a short period of time when applied to a wound bed [39–41]. Recent studies demonstrate the development of an endothelialized dermal equivalent using ASCs [42], but this approach involved differentiation of ASCs to endothelial cells prior to their incorporation into the matrix, which involves the use of growth factors and adds to the overall timeframe in generating the dermal substitute. Our current approach to regenerate skin involves the simultaneous development of an epithelialized, vascularized dermal substitute that is mainly driven by the inherent ability of the ASC to take cue from its surrounding biomatrix. To accomplish this process, we utilized a collagen-PEGylated-fibrin-based bilayered hydrogel. This bilayered hydrogel is designed to overcome the need to predifferentiate cells before they are incorporated into a bioscaffold and is expected to provide a viable environment for host cells, leading to better wound regeneration. Fibrin and collagen have been used for various biomedical and wound healing applications [41, 43]. Fibrinogen-based products are routinely used for clinical purposes since they promote wound healing by initiating early cellular and molecular events essential for tissue connection and angiogenesis [43, 44]. Fibrin was used here in constructing the dermal layer to take advantage of its inherent ability of providing a three-dimensional provisional matrix for dsASCs to promote vasculogenesis when applied to the wound bed. PEGylated-fibrin gels promoted dsASCs differentiation into tubular networks expressing NG2, demonstrating its commitment to develop vascular phenotype lineage, similar to our previous observation with rat ASCs [24]. Within the collagen layer of the hydrogel construct, dsASCs maintained their fibroblast like phenotype, morphology, and expressed alpha smooth muscle actin (*α*SMA). It has been shown previously that *α*SMA is present in the microfilament bundles of pericytes [45] and localizes with CD31 expressed by endothelial cells [28, 46]. Furthermore, dsASCs also expressed STRO-1 (data not show), confirming the stromal cell phenotype commitment within the collagen hydrogel. In this study, we show that dsASCs phenotype can be dictated by the matrix microenvironment as the dsASCs maintain their stromal phenotype in collagen and differentiate into a vascular phenotype in PEGylated fibrin. Therefore, bilayer matrices can direct stem cell phenotypes, as well as act as a template for the creation of vascularized dermal substitute.

Finally, the hypodermis can also be reconstituted using a combination of dsASCs with collagen hydrogels. Traditionally, this layer of skin has been disregarded, as it is not simply replaced through grafting of split- or full-thickness skin. Currently available skin substitutes are also targeted at replacing the epidermis and/or dermis alone with little attention paid to the hypodermis [39, 41]. However, several studies have now demonstrated the beneficial effects of adipose tissue in conjunction with soft-tissue reconstructions [47, 48]. In cases of deep full-thickness burns, replacement of the hypodermis has the obvious advantage of replenishing bulk and diminishing the contour irregularity. It may also have potential benefits of improving dermal skin quality and improved thermoregulation. When dsASCs in the collagen matrix were induced with adipogenic media, they were able to differentiate into adipocytes and showed positive staining with Oil Red O. Within the collagen matrix, dsASCs showed a three-dimensional pattern of oil droplets, and this phenomenon is directly influenced by the surrounding microenvironment. A collagen gel is a prototypical hypodermal substitute; and we speculate that dsASCs, when combined with currently available acellular collagen matrices [41, 49–51], could be similarly differentiated *in situ* using adipogenic inducers for the creation of a hypodermal skin substitute. 

The skin substitute developed in this study, using different natural biomaterials and autologous stem cells, is intended to be used for extensive burn wound regeneration. In the current study, 1 × 10^5^ dsASCs are sufficient to cover a wound with a total surface area of *≈*2 cm^2^, therefore using 1 × 10^6^ dsASCs would allow the coverage of larger, up to 20 cm^2^, surface area wounds. In addition, our collagen-PEGylated-fibrin-based bilayer hydrogel that improves revascularization can be applied in conjunction with FDA approved skin substitutes to improve vascularization, integration, and remodeling of the matrix substitute by the host to heal the wound bed. We have also recently developed a PEGylated-fibrin gel construct, embedded with silver sulfadiazine loaded microspheres that possesses both antimicrobial and angiogenic properties [52]. We envision developing an engineered skin substitute that also possesses antimicrobial properties, since current products cannot be placed on infected wounds due to their lack of infection control.

## 5. Conclusion

The theoretical advantages of replacing all three anatomic layers of missing skin is to improve the quality of grafted skin long term, with need for fewer surgical revisions. While additional studies are needed to demonstrate the feasibility of this approach, we have demonstrated that viable stem cells can be harvested from the excised burn eschar and that, when exposed to the appropriate stimuli, they will differentiate into the various mesenchymal cell types, corresponding to the three layers of skin. When mixed with the appropriate scaffolds and inducers, this source of stem cells can potentially be utilized for immediate wound coverage without the need for cell expansion. Furthermore, this approach will allow for the entire procedure to be performed within the operating room immediately after burn eschar excision for permanent wound coverage. Alternatively, the autologous cells can be stored to be used for secondary revisional procedures. By introducing additional complexity into the coverage of a burn wound, the risks of failure naturally increase. However, the potential benefits of achieving better long-term outcomes combined with fewer operations needed for early coverage or for secondary revisions can easily outweigh that risk. Our bilayer collagen-PEGylated-fibrin hydrogel has the advantage of inducing robust vascularization, facilitates incorporation, and consequently may also lower the risks of infection. Further demonstration of this methodology in a preclinical model is under way.
